# Estimation and Optimization of Tool Wear in Conventional Turning of 709M40 Alloy Steel Using Support Vector Machine (SVM) with Bayesian Optimization

**DOI:** 10.3390/ma14143773

**Published:** 2021-07-06

**Authors:** Mahdi S. Alajmi, Abdullah M. Almeshal

**Affiliations:** 1 Department of Manufacturing Engineering Technology, College of Technological Studies, P.A.A.E.T., P.O. Box 42325, Shuwaikh 70654, Kuwait; 2 Department of Electronics Engineering Technology, College of Technological Studies, P.A.A.E.T., P.O. Box 42325, Shuwaikh 70654, Kuwait; am.almeshal@paaet.edu.kw

**Keywords:** artificial intelligence, tool wear, turning machine, SVM, Bayesian optimisation

## Abstract

Cutting tool wear reduces the quality of the product in production processes. The optimization of both the machining parameters and tool life reliability is an increasing research trend to save manufacturing resources. In the present work, we introduced a computational approach in estimating the tool wear in the turning process using artificial intelligence. Support vector machines (SVM) for regression with Bayesian optimization is used to determine the tool wear based on various machining parameters. A coated insert carbide tool 2025 was utilized in turning tests of 709M40 alloy steel. Experimental data were collected for three machining parameters like feed rate, depth of cut, and cutting speed, while the parameter of tool wear was calculated with a scanning electron microscope (SEM). The SVM model was trained on 162 experimental data points and the trained model was then used to estimate the experimental testing data points to determine the model performance. The proposed SVM model with Bayesian optimization achieved a superior accuracy in estimation of the tool wear with a mean absolute percentage error (MAPE) of 6.13% and root mean square error (RMSE) of 2.29%. The results suggest the feasibility of adopting artificial intelligence methods in estimating the machining parameters to reduce the time and costs of manufacturing processes and contribute toward greater sustainability.

## 1. Introduction

Tool life is one of the main parameters in machining. Tools that wear or fail a comparably lengthy duration life service can lead to a decreased production rate and surface finish capacity [1]. Tool wear is an important parameter in machining as its increase not only increases cutting forces and cutting temperatures but also produces poor finished and inaccurately machined surfaces. Rapid tool wear also increased the lead time spent in the replacement of tools, thereby reducing the production rate [2]. Worn tools reduce the quality of the production and might harm the machine as well as the workpiece. Furthermore, the cutting force might increase, which elevates the temperature and intensifies the tool wear. Breakdown of the tool may result in more significant repercussions like scraping as well as scratching and might cause the workpieces and tool holder to be catastrophic. Increased cutting forces and power consumption, declining dimensional accuracy as well as surface quality are indicators of failure of the tool [3]. However, the turning process has become one of the major metal production processes among metal-cutting procedures and is frequently used for industrial applications in the area of high technology [4]. In the turning process, tool wear occurs because of the contact between the workpiece and cutting tool, which is directly affected by the cutting parameters, cutting forces, tool geometry, power consumption, etc. Feed rate, depth of cut, and cutting speed are the common parameters as described previously [5,6]. Machining parameters are determined through trial and error based on experience and handouts of process planners are expensive and time-consuming [7]. A human process planner selects the correct process parameters based on their expertise or machining tables. In most situations, the traditional and far from ideal specified parameters are present. However, it is important to decide the correct settings during machining. If the machining settings are not adequate, extensive tool wear is seen and surface degradation may arise. The machining variables like progressive tool wear have been shown to have an influence on optimal cutting parameters and only the selection of suitable optimum parameters may lead to excellent machining process performance [8]. Many different studies have described approaches of machine learning (e.g., to assess the tool wear), and the machines used are namely SVR (“support vector regression”), SVM, and ANNs (“artificial neural networks”).

A tool wear predictive model was introduced by Kong et al. [9] based on RVM (“relevance vector machine”) and KPCA_IRBF (“integrated radial basis function-based kernel principal component analysis”). The outcomes demonstrate that KPCA IRBF can lower the RMSE of RVM by over 30% and decrease the confidence interval (CI) width by over 90 percent. Alajmi and Almeshal [10,11,12] proposed different machine learning methods such as ANFIS-PSO, XGBoost-SDA, Gaussian process regression algorithm, and least squares boosting ensemble, and quantum-behaved PSO to solve manufacturing processes (e.g., drilling, turning, and milling).

Jurkovic [13] compared three approaches for machine learning approaches to determine independent output cutting parameters in a high-speed turning mechanism: SVR, ANN, and polynomial (quadratic) regression were utilized. To determine three output machining parameters, the findings demonstrate no substantial performance difference between polynomial regression and SVR. Multilayer perceptron (MLP), used by Twardowski [14], was used for the prediction of tool wear based on mechanical vibration and cutting forces during turning hard steel. Using measurements of cutting force components, the wear projection marginally achieved more excellent performance than using vibration accelerations. The RMSE = 0.045 mm was the accomplished error. This implies that both vibration acceleration and cutting power are equally appropriate for measuring tool wear when processing hard-to-cut materials.

McParland et al. [15] presented the Bayesian Gaussian hierarchical process model to estimate the rates of tool wear for untested experimental scenarios. The results showed that the projected rate of tool wear is non-linear and that the model may suggest trial conditions to improve the tool’s life. In conjunction with the local feature extractor, for long-term prediction, Wang et al. [16] used a recently constructed heterogeneous GRU model. Systematic feature engineering and optimum searching of the hyperparameter optimizes the given model. However, practical investigations on the wear test to prove the superiority and precise MSE and RMSE of the proposed model compared with the most widely explored multi-variate regression prediction model have been carried out. Sheng [17] presented a modeling technique for turning parameters coupled depending on the least cutting-tool wear. With orthogonal trials, the equation between cutting parameters and temperature was achieved, therefore, the cutting parameters coupled were established with minimal wear of the cutter. The impact of cutting parameters on cutting temperature, cutting force, wear mechanism, and tool life as well as surface roughness were explored by Zheng et al. [18]. The most significant impact on cutting force, tool life, and cutting temperature was indicated by the cutting speed, whereas the surface roughness was substantially impacted by feeding rate.

For the control of tool wear, D’Addona et al. [19] used two naturally inspired computing approaches including DBC (“DNA-based computing”) as well as ANN. The results showed that the ANN may determine the tool-wear degree in a series of tool-wear photos processed according to a certain protocol while the DBC may recognize the similarity/unlike degree in the photos processed. Chang et al. [20] investigated the BNN (“backpropagation neural network”) based on iterative gradient convergences for the estimation of tool life by evaluating its stability and convergence.

Iterative convergence estimation techniques include ADAM (adaptive moment), Adadelta (adaptive delta), Adagrad (adaptive gradient), momentum, and SGD (stochastic gradient descent) algorithms. The outcomes of these techniques demonstrate that the best prediction for the BNN model is the convergence of the ADAM gradient for tool wear in all instances. To predict tool wear, Wang et al. [21] introduced a new physics-guided neural network model. As a modeling strategy for the combination of secret data examined by a data-driven model and a physics-based model, a cross-physical data fusion system (CPDF) was introduced, then the data hidden in the unlabeled sample were examined by the physics-based model of tool cutting influenced by semi-supervised learning. The benefit of the proposed approach is that it examines adequate physics and data domain information to remove physical inconsistency from standard data-driven models.

The deep learning network is known as the deep belief network, DBN, which was introduced by Chen et al. [22] to forecast the wear of a cutting tool on the flank. The performance of a DBN was compared against the performance of SVR and ANNs on an MSE basis (“mean-squared error”) and the coefficient of determination (R^2), taking into consideration the data of over 900 tests to determine the superiority of the DBN in forecasting the tool wear. Wu et al. [23] applied BiLSTM (bidirectional long short-term memory) as well as SVD (singular value decomposition) neural network for the projection of tool wear. The results of the experiments revealed that the suggested SVD-BiLSTM model can efficiently estimate the tool wear and provide better prediction than other comparative models.

Shen et al. [24] developed a predictive model by employing innovative techniques of machine learning with a multi-feature multi-model ensemble and dynamic smoothing method with machining parameters (i.e., feed rate, depth of cut as well as cutting speed) as model inputs, where the main characteristics for estimations were therefore generated. With prediction outcomes, the trials revealed great agreement in terms of predictive trends and the precision of the average values of RMSE.

This work proposes the SVM with Bayesian optimization for regression for estimating the nose wear in the turning of 709M40 alloy steel. This research contributes to the investigation of artificial intelligence approaches in estimating the machining parameters to reduce the processing time, resources, and labor. In addition, the research investigates the efficacy of the SVM model with Bayesian optimization in estimating the tool wear in the turning process. Moreover, the research highlights the importance of utilizing artificial intelligence in contributing toward more sustainable manufacturing.

## 2. Materials and Methods

The experimental work provided by the College of Technological Studies was conducted at the workshop on machining at the Department of Manufacturing Engineering Technology. This work aimed to obtain appropriate dependent values in terms of machining parameters from independent input values of machining parameters. A series of turning trials were performed by utilizing a multicoating composed of TiCN + Al_2_O_3_ + TiN deposited by CVD carbide removable inserts as part of a coupled project for optimizing machining operations (SPUN 12 03 12 2025) with four squared working edges [25] for cutting alloy steel-709M40. Insert configurations were 30°, 60°, 0°, 5°, 6°, side approach and approach angles, inclination, clearance, and normal rake, respectively.

Turning experiments were performed in dry conditions using lathe type Harrison 600 with spindle speed range of 10–1800 rpm. The required tool holder type was a CSBPL 2020K 12. The workpiece was a 709M40 alloy steel bars, which were around 410 mm long with a diameter of 100 mm. The workpiece is often supplied in the hardened and tempered condition with a tensile strength ranging from 850–1000 $N/{\mathrm{mm}}^{2}$ due to its good ductility and shock resistance and resistance to wear properties [26]. The treatment condition used for the workpiece was soft annealed. The chemical composition, mechanical, and physical properties of the 709M40 alloy steel is given in Table 1. However, to match the practical rough turning, criterion values of nose wear were considered in this study.

## 3. Methodology

### 3.1. Support Vector Machine with Bayesian Optimization for Regression

A SVM for regression is one of the well-known machine learning methods that was first introduced by Drucker et al. [29]. SVM has been widely adopted for various regression problems and has achieved results with high accuracy when compared to other machine learning models. SVM can provide predictions for small and high dimensional data, data with local minima, and nonlinear problems [30].

SVM is based on a mathematical basis and statistical learning approaches that can improve the generalization capability by employing the structural risk minimization (SRM) principle [29,30].

**Figure 1:**
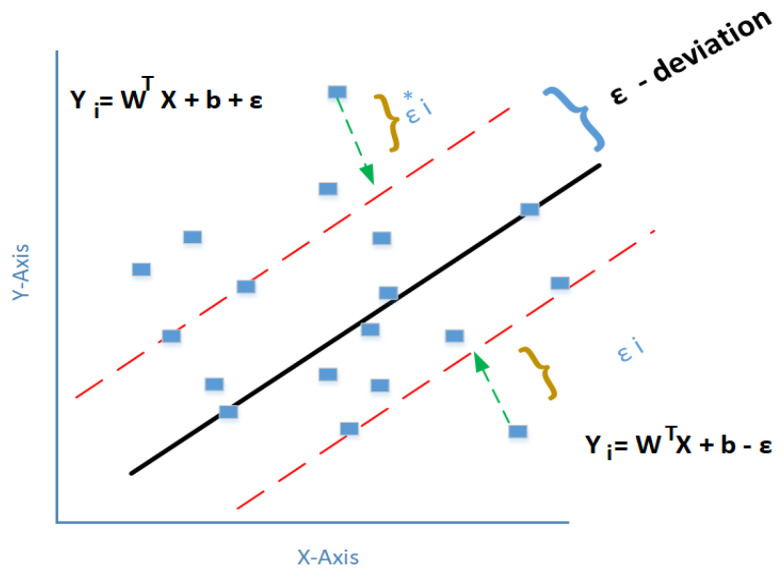
Support vector regression model.

For the training dataset Y = {($a_{1}$, $b_{1}$), ($a_{2}$, $b_{2}$), ($a_{3}$, $b_{3}$), …, ($a_{n}$, $b_{n}$)}, $a_{i}$, $b_{I}$ ∈ R, where $a_{i}$ represents the feature vector of the input sample and $b_{i}$ is the corresponding labels to each sample for *i* = 1, 2, 3, …, *n*. The fundamental concept of SVM is to build a nonlinear map between output and input and then map the input data into the high dimensional feature space from low dimension using kernel as a function. A simple SVM model is shown in Figure 1.

(1) f(a)=w.φ(x)+b

where φ(x) is a nonlinear function in which a low dimension feature input will be converted into high dimensional feature space. In SVM, W contains the coefficients of the data, and b is the learnable constant. The SVM objective function can be formulated as:

(2) $$\begin{array}{c} min\;\\ w,b,\;\varepsilon_{i},\varepsilon \begin{array}{c} {}*\\ i \end{array}\; \end{array}\;\frac{1}{2}||w|{|}^{2}+c\;\overset{n}{\underset{1=1}{\sum }}\left(\varepsilon_{i}+\varepsilon_{i}^{*}\right)\;\;$$

(3) $$subject\;to\{\begin{array}{c} f{\left(a\right)}_{i}-y_{i}\;\leq \varepsilon +\varepsilon_{i}\;\\ \;y_{i}-f\left(a_{i}\right)\leq \varepsilon +\varepsilon_{i}^{*}\\ \varepsilon_{i}\geq 0,\varepsilon_{i}^{*}\geq 0,i=1,2,\ldots ,n. \end{array}\;\;\;$$

In the above equation where $\varepsilon_{i}^{*}$ and $\varepsilon_{i}$ are the upper and lower slack variables. This is subject to ε –deviation $y_{i}-f\left(a_{i}\right)\leq \varepsilon$, the term $\frac{1}{2}||w|{|}^{2}$ is a regularization that improves the generalization of the model.

The regularization constant parameter C determines the trade-off between experiential error and believing risk. In Equation (3), the ε denotes the loss parameter. Equation (2) constraint suggests that the loss will be ignored if the difference between the real and predicted values is less than ε. As shown in Figure 1, the actual value of the data $a_{i}$ is under the ε, where the chance of getting an error is quite a low estimate as zero. On the other hand, if the actual values $b_{i}$ are outside the ε , then the error will be $\varepsilon_{i\;}or\;\varepsilon_{i}^{*}$.

Machine learning models are prone to underfitting and overfitting during a training phase. Thus, to avoid this problem, the regularization term, $\frac{1}{2}||w|{|}^{2}$ as well as c , the training error is minimized [30] Hyperparameters play an important role in fine-tuning the performance of machine learning models [31]. The tuning of hyperparameters can be either a manual or optimized approach. Manual approaches are time-consuming and may not achieve optimal performance. Due to this, various optimization approaches can be integrated into the selection of hyperparameters of machine learning models such as Bayesian optimization, heuristical optimization approaches, random search, and grid search methods. In this research, Bayesian optimization is utilized to optimize the hyperparameters of the SVM model.

### 3.2. Bayesian Optimization

In the Bayesian theory concept, by calculating the objective function of the posterior distribution, which captures the updated belief for the known objective function. For objective function *f(a),* Bayesian optimization constructs a probabilistic model. The model is exploited to predict the unknown next point to evaluate the bounded set A. From the preceding evaluation *f(a)* to make full use of predicted information and is not limited only on the Hessian approximations or local gradient, it can also find the maximum complex of non-convex functions [32].

There are two-parts that play a role with Bayesian optimization. The first one is the Gaussian process prior and the second one is the acquisition function that is used to evaluate the subsequent point by constructing a utility function.

The Gaussian process is an effective and powerful prior distribution over the space smooth function. It is based on a random variable that has no limit (e.g., an infinite number), where any finite random variable is subjective to combined Gaussian distribution [32].

The Gaussian distribution (GP) is expressed as:

(4) $$f\left(a\right)\sim GP\left(\text{µ}\left(a\right),k\left(a,a^{*}\right)\right)$$

where µ(a) is a mean function of a and the $k\left(a,a^{*}\right)$ value data is a covariance function of data $a,a^{*}$.

There are many ways to choose an acquisition function like expected improvement (EI) as well as probability of improvement (PI). The PI function is written as

(5) $$\alpha PI\left(a\right)=\phi \left(ɤ\left(a\right)\right),\left(ɤ\left(a\right)\right)=\frac{f\left(a_{best}\right)-\mu \left(a\right)}{\sigma \left(a\right)}$$

where σ(a) and μ(a) indicate the predictive variance and mean function of the objective function.

Φ(.) and ɤ(.) express the PDF as well as CDF of standard normal distribution. The current best observation is donated by $a_{best}=arg\;max{\;}_{a_{i\;\in a_{1:t}}}f\left(a_{i}\right),$ respectively. In the SVM model, there are three parameters (C, ϵ, σ) to optimize, where C is the penalization coefficient; the kernel parameter is denoted by *σ*, when σ value is larger, the structural risk will be small; and ϵ is the insensitive loss coefficient that controls the gap (width) of the regression function on the insensitive area [33].

## 4. Results and Discussion

**Figure 2:**
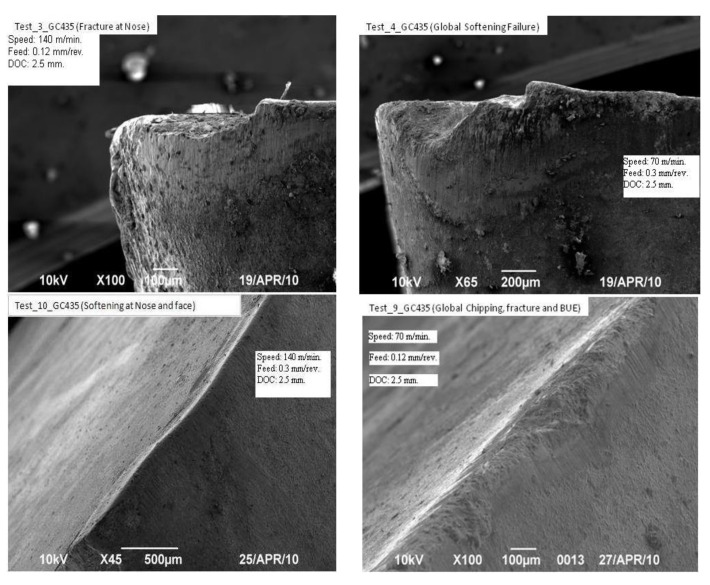
Micrographs of nose wear for different cutting speeds and feed rates.

Table 2 illustrates twenty-four experiments with varied cutting parameters that were carried out under dry conditions. The trials were performed in a single-path double-pass system. Based on the second pass in each pass, the response was computed. This was provided to facilitate considerable wear for a 709M40 steel alloy to take place with 24 experimental trials. Scanning images of the nose wear were measured by field-emission scanning electron microscope (FESEM), as shown in Figure 1. The figure illustrates the micrographs of nose wear for different cutting speeds and feed rates. Nose wear was found to be the dominant wear mechanism than the flank and crater wear [34]. Figure 2 shows a comparison of tool edge performance in three experiments where low, moderate, and high cutting speed are used in association with a moderate feed of 0.2 mm/rev and a depth of 2.25 mm. To match the practical rough turning operations, nose wear was considered as a criteria in the experiment. A Zeiss Gemini SEM 500 field emission scanning electron microscope (FESEM) equipped with an energy-dispersive X-ray (EDX) microanalysis system was utilized to examine the worn tool inserts.

### 4.1. Training Dataset

To train the SVM model for determining the tool wear, data from experiments in the literature were extracted. Table A1 depicts the training data from [5] that was used to train the SVM model on the different feed rate, depth of cut, and cutting speed. It can be seen that the training data had 82 experiments for the CVD coated tool and similarly for the PVD coated tool, it added to a total of 162 experiments for the training dataset. The depth of cut (d), feed rate (f), and cutting speed (V) were considered as the input parameters whereas the wear of the cutting tool insert was considered as the response. The selection of the training dataset was based on the large numbers of experiments presented in [5], where large datasets are preferable in training artificial intelligence algorithms. In addition, the data present the input parameters of interests such as depth of cut, feed rate, and cutting speed with a comparable range, in terms of standard deviation of depth of cut, to our conducted experiment of the validation dataset of Table 2.

**Figure 3:**
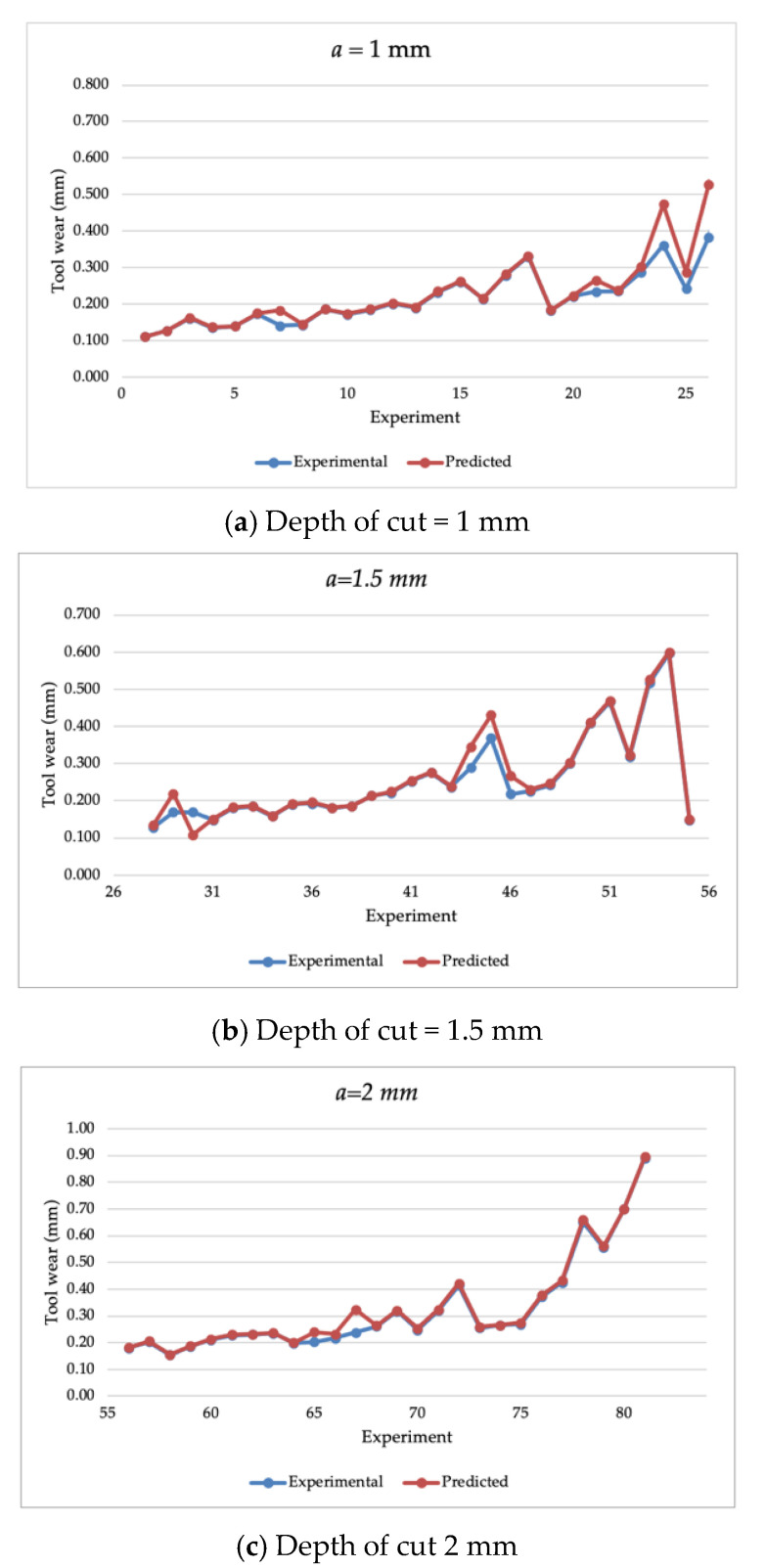
The estimated tool wear of the training dataset parameters in the CVD coated cutting tool with a depth of cut of (**a**) 1 mm, (**b**) 1.5 mm, and (**c**) 2 mm.

**Figure 4:**
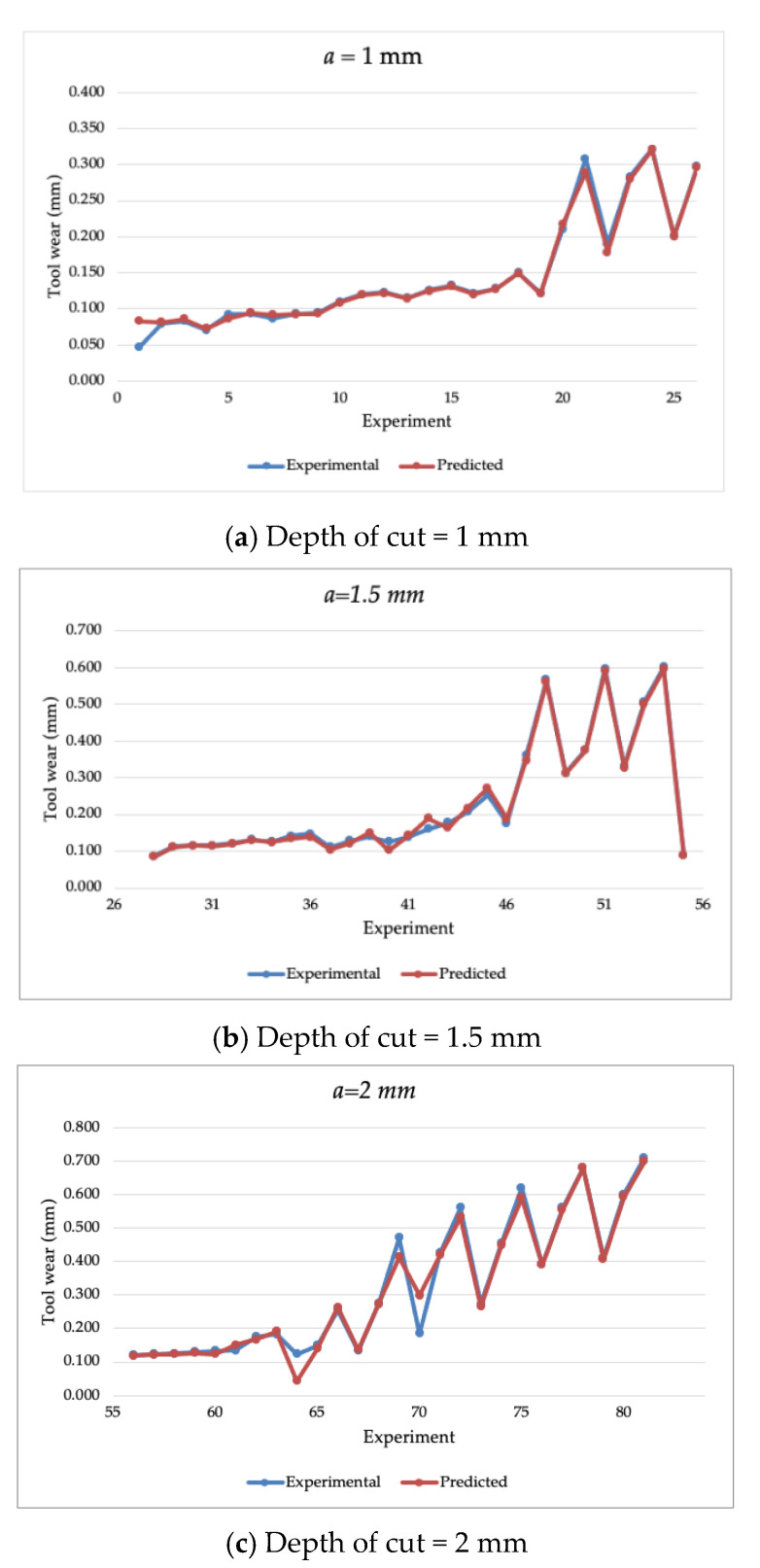
The estimated tool wear of the training dataset of a PVD cutting tool with a depth of cut of (**a**) 1 mm, (**b**) 1.5 mm, and (**c**) 2 mm.

The SVM with the Bayesian optimization model was executed with an acquisition function defined as the probability of improvement with 30 iterations. Figure 3 illustrates the estimation of the tool wear of the CVD-coated cutting tool wear of the training dataset, while Figure 4 presents the estimation of the cutting tool wear of the PVD coated tool wear of the training dataset. It can be observed that the SVM model with Bayesian optimization achieved a high extent of accuracy by closely estimating the experimental training dataset. Table A2 presents the estimated value of the tool wear parameter of each experiment in the training dataset. To further assess the performance of the estimation results, statistical measures were calculated as the MAPE (“mean absolute percentage error”), RMSE (“root mean square error”), and CVRMSE (“coefficient of variation of root mean square error”), which are calculated in Equations (6)–(8), respectively, as:

(6) $$MAPE=\frac{1}{n}\overset{n}{\underset{i=1}{\sum }}\left|\frac{{\hat{y}}_{i}-y_{i}}{y_{i}}\right|\times 100\%\;$$

(7) $$RMSE=\frac{\sqrt{\sum_{i=1}^{n}{\left({\hat{y}}_{i}-y_{i}\right)}^{2}}}{n}$$

(8) $$CVRMSE=\frac{\sqrt{\sum_{i=1}^{n}{\left({\hat{y}}_{i}-y_{i}\right)}^{2}}}{\overset{˘}{y}}$$

where y denotes the experimental data point; $\hat{y}\;$denotes the estimated data point; and $\overset{˘}{y}$ as the average value. Table 3 presented the calculated performance metrics of the SVM model with Bayesian optimization for the training dataset. The model resulted in superior performance in terms of the MAPE, RMSE, and CVRMSE with an average of 5.36%, 2.48%, and 15.9%, respectively.

### 4.2. Validation Dataset

**Figure 5:**
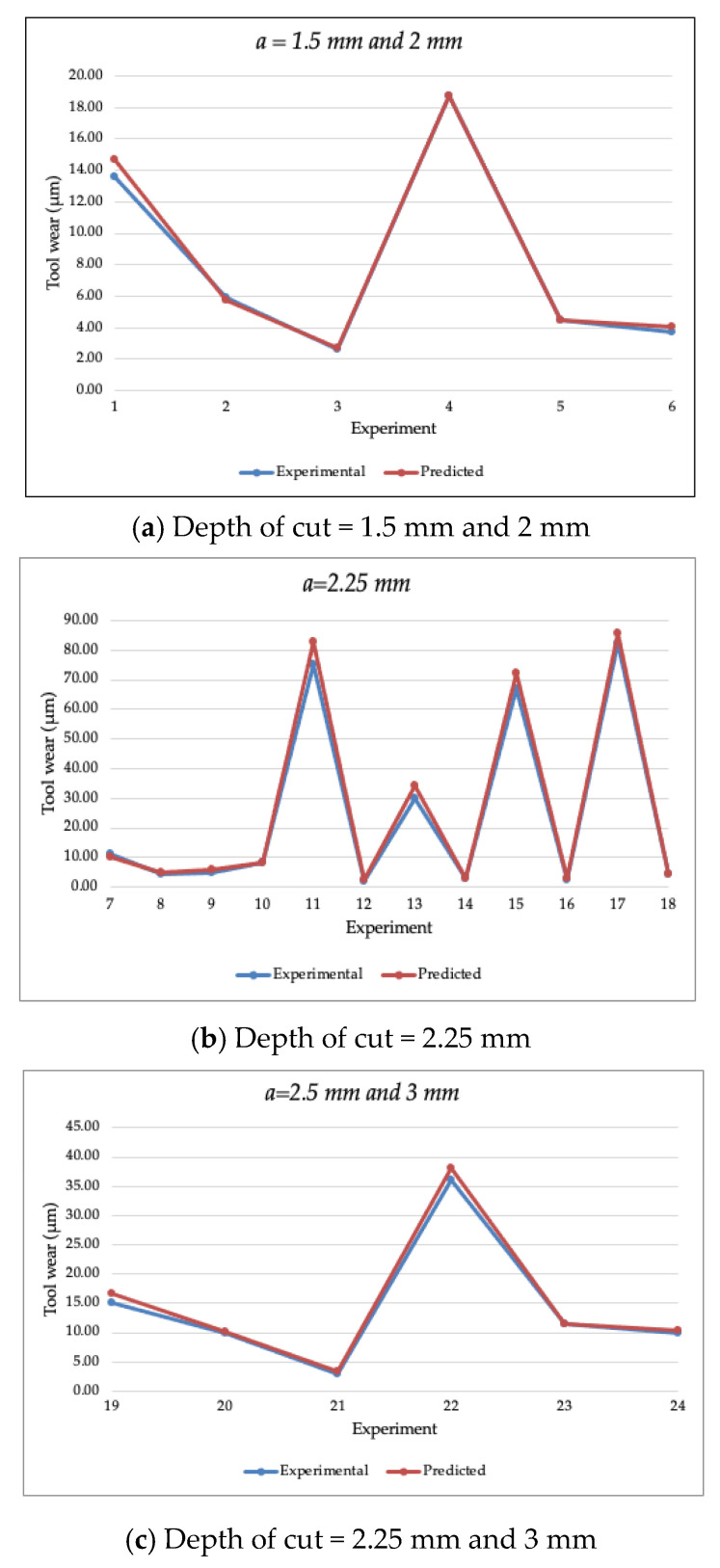
The estimated tool wear of the validation dataset with the various depth of cuts of (**a**) 1.5 mm and 2 mm, (**b**) 2.25 mm, and (**c**) 2.25 mm and 3 mm.

The promising trained model was then exported and used to estimate the cutting tool wear of the validation dataset that was presented in Table 2. Figure 5 illustrates the experimental and predicted data of the cutting tool wear of the validation dataset with various depths of cuts of 1.5 mm, 2 mm, 2.25 mm, 2.5 mm, and 3 mm. The estimated data points by the SVM model with Bayesian optimization closely matched the experimental data points of the validation dataset. Table 4 depicts the estimated individual values of the cutting tool wear parameter for each experiment in the validation dataset. The performance metrics of the SVM model with Bayesian optimization on the validation dataset were calculated and are presented in Table 5. It can be noted that the proposed SVM model achieved a high extent of accuracy in estimating the cutting tool wear parameter of the validation dataset with a MAPE of 6.13%, RMSE of 2.29%, and CVRMSE of 9.02%. The results validate the model performance and the feasibility of estimating the cutting tool wear computationally based on the previous training dataset and thus can reduce the experimental work and resources.

## 5. Conclusions

This research explored the performance of the artificial intelligence-based estimation approach of tool wear in a turning process of 709M40 alloy steel. Support vector machine for regression with Bayesian optimization model was employed to estimate the tool wear value based on the depth of cut and cutting speed as well as feed rate inputs. The proposed model was trained on a training dataset of 162 data points and was then used to determine the tool wear of our validation dataset. The reported results suggest the feasibility of the SVM model in estimating the tool wear with high accuracy of MAPE of 6.13%, RMSE of 2.29%, and CVRMSE of 9.02% for the validation dataset. The estimated values closely match the conducted experimental tool wear values. The approach was validated for the 709M40 alloy steel based on depth of cut, feed rate, and cutting speed for a certain range of values. For other materials with a different range of input values, it would require retraining the algorithm using the transfer learning approach. The proposed approach contributes more toward saving the cost, time, and labor in the manufacturing process. Moreover, adopting artificial intelligence estimation methods saves resources and enables more efficient and sustainable manufacturing processes.

## Figures and Tables

**Table 1:** Chemical composition, mechanical, and physical properties of the 709M40 alloy steel [27,28].

Composition	(Wt%)	Mechanical Properties	Values
C	0.44	Tensile strength $N/{\mathrm{mm}}^{2}$	850–1000
$C_{r}$	0.99	$\mathrm{Yield}\;\mathrm{Strength}\;N/{\mathrm{mm}}^{2}$	650
Mo	0.25	Hardness (HB)	248–302
Mn	0.85	Elongation %	13
S	<0.04	Impact test (KV)	50
P	<0.04	Density $\left(\frac{\mathrm{Kg}}{{\mathrm{dm}}^{3}}\right)$	421

**Table 2:** The experimental data.

Experiment Trial	V [m/min]	f [mm/rev]	d [mm]	Nw [µm/min]
T1	102.67	0.2	1.5	13.6
T2	103.4	0.2	1.5	5.88
T3	72.35	0.12	2	2.65
T4	145	0.3	2	18.75
T5	148.22	0.12	2	4.48
T6	72.69	0.3	2	3.7
T7	104	0.2	2.25	11
T8	104.72	0.2	2.25	4.6
T9	103.67	0.2	2.25	5
T10	103.06	0.2	2.25	8.33
T11	206	0.2	2.25	75
T12	50	0.2	2.25	2.14
T13	101.71	0.6	2.25	30
T14	104.15	0.06	2.25	2.91
T15	206	0.2	2.25	66.7
T16	50.36	0.2	2.25	2.5
T17	103.55	0.6	2.25	82.41
T18	104.8	0.06	2.25	4.55
T19	145.89	0.12	2.5	15
T20	72	0.3	2.5	10
T21	72.32	0.12	2.5	2.97
T22	144.72	0.3	2.5	36.14
T23	104.66	0.2	3	11.54
T24	103.75	0.2	3	10

**Table 3:** Statistical performance metrics of the SVM model with Bayesian optimization for the training dataset.

Metric	Training Dataset	
	PVD Coated	CVD Coated
MAPE %	5.630	5.096
RMSE %	1.936	3.040
CVRMSE %	11.619	20.326

**Table 4:** The estimated cutting tool wear of the validation dataset.

Data	Actual Nw [μm/m]	Estimated Nw [μm/m]
1	13.60	14.683
2	5.88	5.731
3	2.65	2.736
4	18.75	18.691
5	4.48	4.492
6	3.70	4.046
7	11.00	10.385
8	4.60	4.681
9	5.00	5.684
10	8.33	8.463
11	75.00	82.834
12	2.14	2.309
13	30.00	33.981
14	2.91	2.994
15	66.70	72.202
16	2.50	2.928
17	82.41	85.384
18	4.55	4.607
19	15.00	16.600
20	10.00	10.211
21	2.97	3.354
22	36.14	38.199
23	11.54	11.472
24	10.00	10.354

**Table 5:** Statistical performance metrics of the SVM model with Bayesian optimization for the validation dataset.

Metric	Validation Dataset
MAPE %	6.13
RMSE %	2.29
CVRMSE %	9.02

## Data Availability

The data presented in this study are available on request from the corresponding author.

## Nomenclature

*V*	Cutting Speed (m/min)
*f*	Feed Rate (mm/rev)
*d*	Depth of Cut (mm)
*Ra*	Surface Roughness (µm/m)
MAE	Mean Root Square Error
RMSE	Root Mean Square Error
*Nw*	Nose Tool Wear (µm/min)
CVRMSE	Coefficient of the Variation of the Root Mean Square Error

## Appendix A

**Table A1:** Training data of various cutting parameters to train the SVM model [5].

Exp No.	Cutting Parameters			Cutting Length	CVD Coated Tool		PVD Coated WC	
	*d* [mm]	*V* [m/min]	*f* [mm/rev]	*L* [mm]	*Ra* [μ/m]	Nw [μ/min]	*Ra* [ μ/m]	Nw [μ/min]
1	1	30	0.052	40	1.82	0.110	1.32	0.047
2	1	30	0.052	80	1.94	0.127	1.54	0.079
3	1	30	0.052	120	2.12	0.16	1.68	0.083
4	1	30	0.104	40	2.15	0.135	2	0.07
5	1	30	0.104	80	2.18	0.139	2.4	0.092
6	1	30	0.104	120	2.48	0.173	2.6	0.093
7	1	30	0.162	40	3.3	0.14	3.72	0.086
8	1	30	0.162	80	3.6	0.143	4.1	0.093
9	1	30	0.162	120	3.8	0.186	4.18	0.094
10	1	60	0.052	40	1.76	0.172	1.3	0.11
11	1	60	0.052	80	1.9	0.184	1.45	0.12
12	1	60	0.052	120	2.04	0.201	1.5	0.123
13	1	60	0.104	40	1.9	0.189	1.76	0.115
14	1	60	0.104	80	2.1	0.232	1.98	0.126
15	1	60	0.104	120	2.3	0.261	2.16	0.132
16	1	60	0.162	40	3.5	0.214	4.12	0.121
17	1	60	0.162	80	3.85	0.279	4.22	0.128
18	1	60	0.162	120	4.1	0.330	4.34	0.15
19	1	90	0.052	40	1.4	0.183	1.28	0.121
20	1	90	0.052	80	1.6	0.221	1.4	0.21
21	1	90	0.052	120	2.3	0.234	1.68	0.308
22	1	90	0.104	40	2.25	0.235	1.94	0.19
23	1	90	0.104	80	2.3	0.287	2.24	0.283
24	1	90	0.104	120	2.7	0.361	2.44	0.321
25	1	90	0.162	40	4	0.242	4.68	0.201
26	1	90	0.162	80	4.3	0.383	4.74	0.297
27	1	90	0.162	120	4.9	0.586	5.08	0.38
28	1.5	30	0.052	40	2.04	0.128	2.02	0.086
29	1.5	30	0.052	80	2.1	0.169	2.2	0.112
30	1.5	30	0.052	120	2.15	0.17	2.4	0.116
31	1.5	30	0.104	40	2.18	0.148	2.24	0.115
32	1.5	30	0.104	80	2.2	0.18	2.32	0.121
33	1.5	30	0.104	120	2.58	0.185	2.72	0.132
34	1.5	30	0.162	40	3.64	0.158	4.16	0.126
35	1.5	30	0.162	80	4.24	0.19	4.32	0.142
36	1.5	30	0.162	120	4.5	0.193	4.4	0.148
37	1.5	60	0.052	40	1.82	0.181	1.64	0.111
38	1.5	60	0.052	80	1.98	0.186	1.7	0.129
39	1.5	60	0.052	120	2.06	0.214	1.86	0.14
40	1.5	60	0.104	40	2.08	0.221	2	0.126
41	1.5	60	0.104	80	2.15	0.252	2.26	0.138
42	1.5	60	0.104	120	2.47	0.276	2.4	0.16
43	1.5	60	0.162	40	4.16	0.237	4.18	0.178
44	1.5	60	0.162	80	4.32	0.289	4.4	0.207
45	1.5	60	0.162	120	4.75	0.369	4.52	0.253
46	1.5	90	0.052	40	2.4	0.218	1.94	0.177
47	1.5	90	0.052	80	2.58	0.226	2	0.36
48	1.5	90	0.052	120	2.6	0.243	2.06	0.567
49	1.5	90	0.104	40	2.47	0.3	2.2	0.313
50	1.5	90	0.104	80	2.54	0.409	2.36	0.376
51	1.5	90	0.104	120	3.16	0.466	3.02	0.597
52	1.5	90	0.162	40	4.16	0.318	4.22	0.331
53	1.5	90	0.162	80	4.78	0.517	4.6	0.507
54	1.5	90	0.162	120	5.1	0.597	5.26	0.602
55	2	30	0.052	40	2.38	0.147	2.4	0.09
56	2	30	0.052	80	2.39	0.18	2.45	0.12
57	2	30	0.052	120	2.6	0.203	2.8	0.123
58	2	30	0.104	40	2.4	0.154	2.65	0.126
59	2	30	0.104	80	2.52	0.186	2.76	0.13
60	2	30	0.104	120	2.64	0.210	2.92	0.134
61	2	30	0.162	40	4.12	0.227	4.46	0.135
62	2	30	0.162	80	4.45	0.23	4.98	0.175
63	2	30	0.162	120	4.65	0.235	5.06	0.183
64	2	60	0.052	40	1.98	0.199	2.25	0.123
65	2	60	0.052	80	2.1	0.203	2.3	0.15
66	2	60	0.052	120	2.45	0.216	2.44	0.256
67	2	60	0.104	40	2.46	0.238	2.68	0.133
68	2	60	0.104	80	2.62	0.261	2.89	0.275
69	2	60	0.104	120	2.84	0.318	3.01	0.47
70	2	60	0.162	40	4.65	0.248	4.68	0.184
71	2	60	0.162	80	4.7	0.32	5.1	0.425
72	2	60	0.162	120	4.76	0.415	5.56	0.56
73	2	90	0.052	40	2.4	0.255	2.3	0.273
74	2	90	0.052	80	2.42	0.265	2.38	0.454
75	2	90	0.052	120	2.68	0.269	2.75	0.620
76	2	90	0.104	40	2.54	0.372	2.92	0.390
77	2	90	0.104	80	2.7	0.425	3.24	0.56
78	2	90	0.104	120	3.2	0.650	3.45	0.680
79	2	90	0.162	40	4.98	0.555	5.34	0.412
80	2	90	0.162	80	5.02	0.7	5.55	0.601
81	2	90	0.162	120	5.4	0.891	5.92	0.710

**Table A2:** Estimated cutting tool wear parameter of the training dataset.

Exp Num	CVD Coated		PVD Coated	
	Experimental	SVM	Experimental	SVM
	Nw [μ/m]	Nw [μ/m]	Nw [μ/m]	Nw [μ/m]
1	0.110	0.110	0.047	0.083
2	0.127	0.128	0.079	0.082
3	0.16	0.163	0.083	0.086
4	0.135	0.137	0.07	0.072
5	0.139	0.140	0.092	0.086
6	0.173	0.175	0.093	0.094
7	0.14	0.182	0.086	0.092
8	0.143	0.145	0.093	0.092
9	0.186	0.186	0.094	0.093
10	0.172	0.173	0.11	0.109
11	0.184	0.186	0.12	0.119
12	0.201	0.203	0.123	0.121
13	0.189	0.191	0.115	0.114
14	0.232	0.236	0.126	0.125
15	0.261	0.263	0.132	0.131
16	0.214	0.215	0.121	0.120
17	0.279	0.282	0.128	0.127
18	0.330	0.332	0.15	0.149
19	0.183	0.184	0.121	0.120
20	0.221	0.223	0.21	0.217
21	0.234	0.266	0.308	0.289
22	0.235	0.237	0.19	0.178
23	0.287	0.303	0.283	0.280
24	0.361	0.473	0.321	0.320
25	0.242	0.288	0.201	0.200
26	0.383	0.528	0.297	0.296
27	0.586	0.687	0.38	0.377
28	0.128	0.135	0.086	0.085
29	0.169	0.220	0.112	0.111
30	0.17	0.108	0.116	0.116
31	0.148	0.150	0.115	0.114
32	0.18	0.183	0.121	0.119
33	0.185	0.186	0.132	0.129
34	0.158	0.160	0.126	0.124
35	0.19	0.191	0.142	0.134
36	0.193	0.196	0.148	0.139
37	0.181	0.181	0.111	0.104
38	0.186	0.186	0.129	0.121
39	0.214	0.214	0.14	0.151
40	0.221	0.224	0.126	0.103
41	0.252	0.255	0.138	0.143
42	0.276	0.276	0.16	0.190
43	0.237	0.239	0.178	0.163
44	0.289	0.346	0.207	0.217
45	0.369	0.431	0.253	0.272
46	0.218	0.267	0.177	0.189
47	0.226	0.230	0.36	0.348
48	0.243	0.247	0.567	0.561
49	0.3	0.304	0.313	0.311
50	0.409	0.411	0.376	0.376
51	0.466	0.469	0.597	0.590
52	0.318	0.322	0.331	0.327
53	0.517	0.526	0.507	0.501
54	0.597	0.600	0.602	0.596
55	0.147	0.149	0.09	0.088
56	0.18	0.182	0.12	0.119
57	0.203	0.205	0.123	0.121
58	0.154	0.155	0.126	0.124
59	0.186	0.188	0.13	0.128
60	0.210	0.213	0.134	0.123
61	0.227	0.231	0.135	0.151
62	0.23	0.231	0.175	0.167
63	0.235	0.236	0.183	0.191
64	0.199	0.199	0.123	0.044
65	0.203	0.240	0.15	0.140
66	0.216	0.232	0.256	0.263
67	0.238	0.324	0.133	0.137
68	0.261	0.265	0.275	0.272
69	0.318	0.322	0.47	0.413
70	0.248	0.253	0.184	0.299
71	0.32	0.324	0.425	0.419
72	0.415	0.421	0.56	0.531
73	0.255	0.259	0.273	0.265
74	0.265	0.266	0.454	0.448
75	0.269	0.274	0.620	0.590
76	0.372	0.378	0.390	0.390
77	0.425	0.432	0.56	0.553
78	0.650	0.660	0.680	0.680
79	0.555	0.561	0.412	0.407
80	0.7	0.701	0.601	0.592
81	0.891	0.895	0.710	0.699
